# In-patient/out-patient detoxification is highly effective in Medication Overuse Headache: report from a multicentric, multinational study

**DOI:** 10.1186/1129-2377-1-S14-P226

**Published:** 2013-02-21

**Authors:** C Tassorelli, R Jensen, M Allena, R De Icco, Z Katsarava, M Lainez, JA Leston, R Fadic, G Nappi

**Affiliations:** 1 IRCCS Neurological Institute C. Mondino Foundation, Italy; 2 Danish Headache Centre, Dept. of Neurology, Glostrup Hospital, Denmark; 3 Department of Neurology, University of Essen, Germany; 4 Fundación de la Comunidad Valenciana, Spain; 5 Fundacion para la Lucha contra las Enfermedades Neurologicas de la Infanzia, Argentina; 6 Pontificia Universidad Catolica de Chile, Chile

## Introduction

Medication overuse headache (MOH) is a common and disabling disorder, potentially treatable but with a high rate of early relapse. Detoxification from the overused drug(s) is rationally and ethically considered as the first and main step in the management of MOH patients, however consensus protocols as well as multicenter studies confirming the efficacy of detoxification are lacking in the literature. The aim is to propose and test on large population a consensus protocol for managing MOH.

## Methods

A consensus protocol for the management of MOH was devised by an expert group. The protocol was based on consolidated clinical expertise and publication records of the members of the group and it foresaw in-patient and/or out-patient detoxification associated with prophylactic treatment and regular follow-up visits over a period of 6 months. The protocol was tested in 6 Centres from Europe and Latin America, which enrolled a total of 387 MOH subjects (313 F, 74 M).

## Results

A marked reduction was observed in both outcome measures was observed already during the first month and tended to improve over the following months. Headache days/month: Baseline 23.2, M1 13.7, M2 11.6, M3 10.6, M4 10.6, M5 10.3, M6 10.2 (p<0.0001 at all time points vs Baseline). Numbers of days of drug intake/ month: Baseline 23.2, M1 11.0, M2 10.2, M3 9.7, M4 9.6, M5 9.8, M6 9.7 (p<0.0001 at all time points vs Baseline). Notably, out-patient detoxification was also effective, perfoming a little less than in-patient detoxification only at M1 in the two otucome measures considered (Days of headache: in-patients 12.0, out-patients 14.7, p<0.03 days of intake: in-patients 7.3, out-patients 12.6, p<0.0001).

## Conclusion

The proposed protocol proved effective in reducing headache days and days of symptomatic drug intake in a large population of MOH sufferers distributed in different clinical and geographical settings. These findings confirm the efficacy and the usability worldwide of a consensus protocol for MOH management.

